# Prognostic Significance of Potential Immune Checkpoint Member HHLA2 in Human Tumors: A Comprehensive Analysis

**DOI:** 10.3389/fimmu.2019.01573

**Published:** 2019-07-15

**Authors:** Ben Wang, Zhujie Ran, Mengmeng Liu, Yunsheng Ou

**Affiliations:** ^1^Department of Orthopedics, The First Affiliated Hospital of Chongqing Medical University, Chongqing, China; ^2^School of Public Health and Community Medicine, Chongqing Medical University, Chongqing, China; ^3^Graduated School of Anhui University of Traditional Chinese Medicine, Hefei, China

**Keywords:** HHLA2, immune checkpoint, B7, PD-1, expression profiling

## Abstract

Immunological checkpoint inhibitors have been immensely successfully applied in the treatment of cancer, however, a portion of tumor patients can't benefit from checkpoint therapy. The low PD-1/CTLA-4 positive rate and involvement of multiple immunosuppressive pathways are thought to be one of the reasons for treatment failure in non-responding patients. A new immune checkpoint molecule, HHLA2, which was widely expressed in PD-1 negative human tumors, may be a promising target for the improvement of recent immune therapy. Yet, the prognostic value and transcriptional regulatory mechanisms of HHLA2 remains unclear. In this study, we aimed to evaluate the prognostic value and transcriptional regulation mechanism of HHLA2 according to clinical and experimental data from multiple databases, including cBioPortal, TCGA, Cistrome, TIMER, Oncomine, Kaplan-Meier, GeneXplain. It was found that the expression of HHLA2 was significantly elevated in renal tumors, and significantly decreased in colorectal tumors. Pan-cancer survival analysis indicates that HHLA2 was an independent prognostic factor in 9/20 of human cancers. Especially in renal clear cell carcinoma (*P* = 3.0E-7). Through plotting survival curve in Kaplan-Meier Plotter, it was found that hypomethylation of HHLA2 DNA was a favorable prognostic factor for KIRC patients. Yet, the copy number variant of HHLA2 was not significantly correlated with the overall survival of KIRC patients. Finally, by analyzing the motif of HHLA2 co-expression genes, we identified 15 transcription factors that may be involved in the regulation of the HHLA2 co-expression network. Among these transcription factors, BATF in B lymphocyte and SMAD in monocyte were confirmed to be able to directly bind to HHLA2 DNA according to chip-seq experimental data from Cistrome database.

## Introduction

Recently, immunotherapy has shown remarkable therapeutic effects in anti-tumor therapy. Among them, the immune checkpoint inhibitors blocking the immunosuppressive receptor of T cells is an effective solution (1). In the opinion of recent studies, the interaction between the B7 and CD28 family plays a central role in regulating T lymphocyte function. The B7-1/B7-2 molecule on the surface of the cell membrane of antigen presenting cell (APC) binds to the CD28 molecule on the surface of T lymphocyte, providing an initial costimulatory signal for the activation of T lymphocyte. After this costimulatory signal, coinhibitory molecule (such as CTLA-4 and PD-1) of CD28 family on the surface of T lymphocyte binds to B7-1/B7-2 to inhibit T lymphocyte activation (2, 3), this interaction mediated T lymphocyte co-stimulation and co-suppression lay the foundation of the regulation of anti-tumor immune responses (4, 5).

Over the past decade, a series of studies have revealed the important immune function of other molecules belonging to B7 and CD28 families, including B7h/ICOS (6), PD-L1/PD-L2/PD-1 (7), B7-H3 (8). Among them, PD-1/PD-L1 inhibitors have achieved great success in clinical trials (9), which has rapidly changed the treatment landscape for non-small-cell lung cancer (NSCLC) (10). Two FDA-approved PD-L1 inhibitors are being evaluated for the first-line treatment of NSCLC (11–14). However, since PD-1 is only expressed in part of NSCLC, finding new broader expressed immune checkpoint will be important for improving the response rate of immunotherapy (15).

Human endogenous retro virus-H Long repeat-associating 2 (HHLA2) is a newly discovered immune checkpoint molecule belonging to the B7-CD28 family (16, 17). Previous studies confirmed that it participates in the regulation of T-lymphocyte function, but previous studies didn't reach an agreement on the function of HHLA2. The first reported high-quality research concluded that HHLA2 (B7-H5) is a costimulatory molecule that acts to promote T lymphocyte proliferation and secret related cytokines by binding to CD28H receptors on T lymphocyte (16). Subsequent research provides a different conclusion of HHLA2 function, Zhao et al. (17) reported that HHLA2 may be a co-inhibitory ligand for T lymphocyte, which inhibits the anti-tumor function of T lymphocyte. In addition, the heterogeneity of HHLA2 function was also observed in the co-culture experiment, the T lymphocytes from different donors showed a different response to the HHLA2 protein in cytokine production (17). This contradictory phenomenon is interesting, but also confusing for the further researches on HHLA2 (18–21).

To contribute to the understanding of these discrepancies, we investigated the expression profiling and prognostic value of HHLA2 in human cancer according to multiple public databases and investigated what transcription factor may be associated with the dysregulation of HHLA2 in KIRC, our finding may be helpful for the further study on HHLA2.

## Materials and Methods

### Expression Profiling of HHLA2 in Human Cancers

Two large-scale databases (GEPIA, Oncomine) was used to explore the expression pattern of HHLA2 in human cancers. Gene expression profiling interactive analysis (GEPIA) (http://gepia.cancer-pku.cn) is an interactive web server for analyzing the RNA sequencing expression data of 9,736 tumors and 8,587 normal samples from TCGA and GTEx projects (22). The Oncomine datasets (23) (http://www.oncomine.org) is a web application for bioinformatics services that includes 715 independent data sets and 86,733 samples that provide greater scale, high quality, consistency, a standardized analytical method for gene expression profile analysis.

### Pan-Cancer Survival Analysis

The Kaplan-Meier plotter (24) (http://kmplot.com//analysis) is capable of assessing the prognostic effect of 54,675 genes on using 10,461 cancer samples. To investigate the prognostic role of HHLA2 in human cancers, the Kaplan-Meier Plotter was used to determine the prognostic significance. The forest plot was constructed by RevMan software (25). UCSC Xena browser (26, 27) was used for evaluating the correlation between expression level of HHLA2 mRNA, methylation status, copy number variant of HHLA2 DNA and the overall survival time in kidney clear cell carcinoma.

To validate the prognostic role of HHLA2 in KIRC, we carefully conducted searches in public data platforms including the GEO database and the ArrayExpress database. The datasets meeting following criteria were used as validation cohorts: Datasets with survival associated clinical information. Datasets from human tumor samples. The genechip used in the datasets includes the probe of HHLA2. After detailed and careful searching, two independent clinical cohorts (GSE40435, GSE22541) containing tumor staging information were used for validation.

### Potential Transcription Regulatory Mechanism of HHLA2

The cBioPortal for Cancer Genomics (http://www.cbioportal.org) provides a Web resource analyzing, visualizing genomics data. The cBioPortal was used for extracting co-expression genes of HHLA2 in KIRC (28, 29). The geneXplain platform (http://genexplain.com/transfac) is a tool for a broad range of bioinformatics applications. Motif enrichment function in this platform was used for the identification of potential transcription factors of co-expression gene network of HHLA2 with the TRANSFAC database (30–32). TFmapper (33) is used to identify whether transcription factors bind directly to the HHLA2 promoter according to cistrome database. The Cistrome data browser (34, 35) integrates human and mouse cis-regulatory experiment information from ChIP-seq, DNase-seq, and ATAC-seq chromatin analysis. The miRWalk (36, 37), miRDB (38, 39), TargetScan (40) are comprehensive online resource for miRNA and target predictions. They were used to identify potential microRNAs. The common part of three predictions results was regarded as potential microRNAs targeting on HHLA2 mRNA.

### Functional Annotation of Co-expression Gene Network of HHLA2

Kyoto Encyclopedia of Genes and Genomes (41) (KEGG) and gene ontology (42) (GO) is a commonly used database of functional annotations for gene list. MetaScape is an excellent integrated analytics platform that integrates multiple annotation datasets. The functional enrichment analysis of this study was applied by this platform to understand this function of HHLA2 co-expression genes (43).

## Result

### The Expression Level of HHLA2 Was Significantly Elevated in Renal Cancers and Decreased in Colorectal Cancers

**Figure 1:**
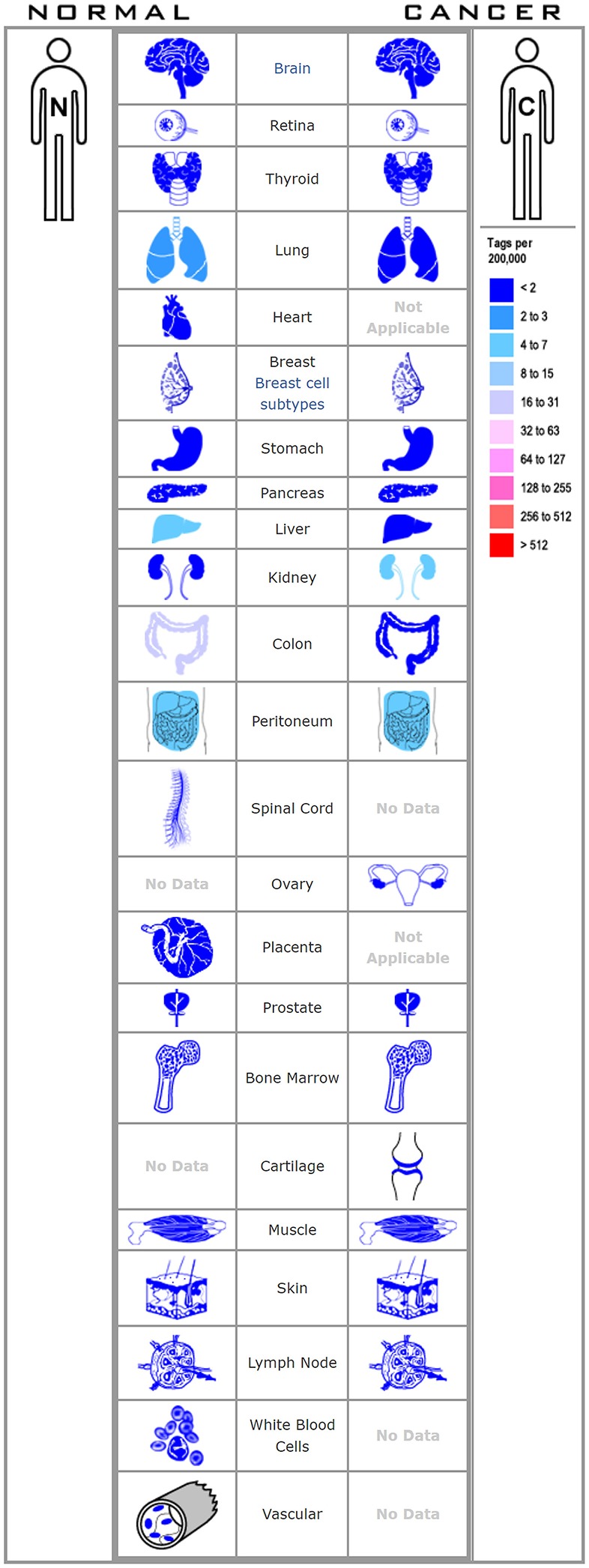
The figure shows the distribution of HHLA2 in various human tissues.

**Figure 2:**
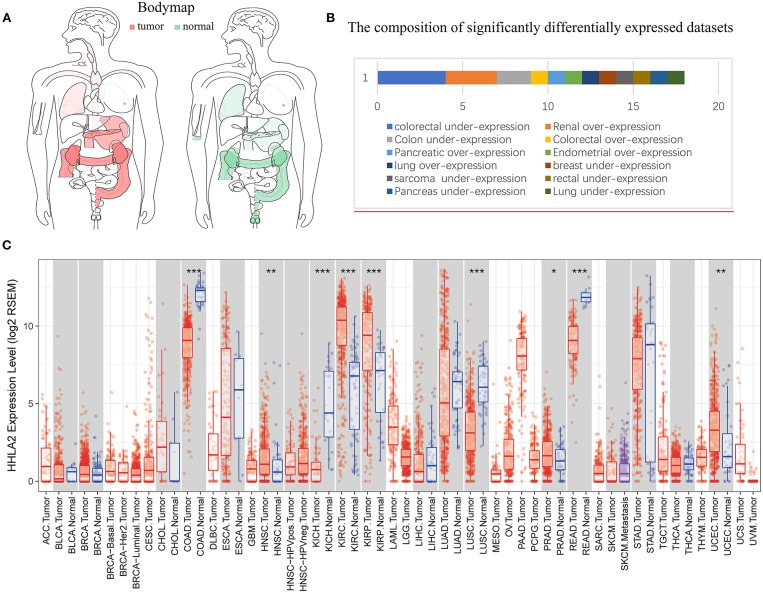
Pan-cancer expression profiling analysis of HHLA2. **(A)** Interactive body map of HHLA2 mRNA expression constructed by GEPIA (TCGA), the darker color corresponds to the higher gene expression level. Red means the median expression of HHLA2 from tumor samples, and the green is from normal samples. **(B)** The length of the bar corresponds to the number of studies show significant over or under expression of HHLA2 vs. normal tissue (the number of significantly differently expressed GEO clinical cohorts). **(C)** The boxplot shows the pan-cancer expression profiling of HHLA2 in human cancers. The below row refers to the standard abbreviations of tumor in TCGA. The color refers to the tumor (red) or normal (blue). *P*-value Significant Codes: 0 ≤ ^***^ < 0.001 ≤ ^**^ < 0.01 ≤ ^*^ < 0.05 ≤. < 0.1.

The Cancer Genome Atlas (TCGA) datasets is a comprehensive database containing 11,000 patient samples. GEPIA website (TCGA data) and SAGE (Figure 1) were used to draw a body map to show the expression distribution of HHLA2 in human tissue (Figure 2A). Then, the pan-cancer expression profiling of HHLA2 was visualized according to TCGA data by TIMER platform (Figure 2C). It was found that HHLA2 is widely expressed in multiple human tissues, and it abounds in renal, colorectal tissue. Next, we investigated whether the expression of HHLA2 is elevated in human cancer compared with normal tissue. Using Oncomine (23) database(*P* < 10E-4, fold Change > 2), which contains 715 datasets and 86,733 samples, we found that HHLA2 is over-expressed in the 7/134 human tumor datasets, and under-expressed in 10/134 datasets (Figure 2B). The most common over-expression cancer type in this study is renal cancer, the most common under-expression cancer type is colorectal cancers.

### Pan-Cancer Survival Analysis of HHLA2 mRNA Expression Revealed a Unique Prognostic Role of HHLA2 in Renal Clear Cell Carcinoma

**Figure 3:**
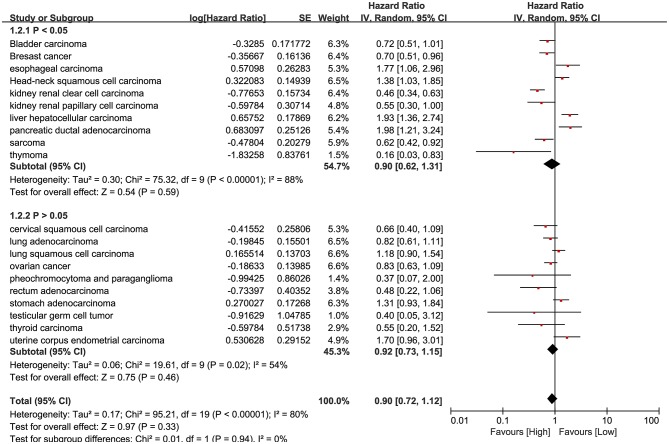
Forest plot shows the result of pan-cancer survival analysis. The upper subgroups are composed of studies whose *P*-value is <0.05, the below subgroups are more than 0.05.

RNA-Seq data of 7,462 samples from Kaplan Meier plotter datasets (24) was used for pan-cancer survival analysis. The significant prognostic significance of HHLA2 was determined in 10/20 tumors in this pan-cancer analysis (Supplementary Table 1). Overview of these results was shown in Figure 3, there is an obvious heterogeneity between different cancer types.

Our result shows that it is hard to determine whether the prognostic role of HHLA2 is favorable or unfavorable. However, in the result of pan-cancer survival analysis, we observed a unique and evident prognostic role of HHLA2 in kidney clear cell carcinoma comparing to other human cancers.

**Figure 4:**
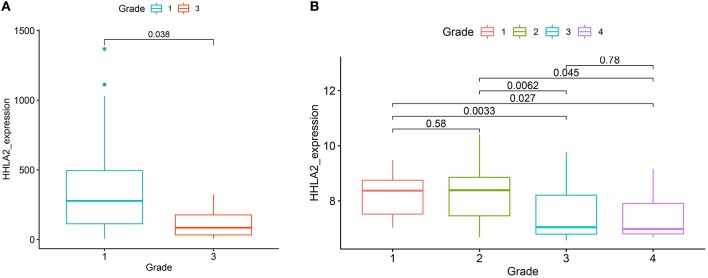
The boxplot shows the correlation between the expression of HHLA2 and the pathological grade in KIRC (left:from GSE22541, right: GSE40435). The label at the top of the picture corresponds to the pathological stage of the patient.

We further extended these results using two independent KIRC cohorts from the GEO database (GSE40435, GSE22541). All of these cohorts showed that the expression of HHLA2 was higher in low pathological grade than the high pathological grade (Figure 4). This was consistent with our result that overexpression of HHLA2 was a protective factor for KIRC patients.

Combing with the result of expression profiling analysis, HHLA2 abounds in human normal and malignant tissue, however only differentially expressed in some tumor types (KIRC, COAD etc.) (Supplementary Table 1), the subsequent studies will focus on the unique role of HHLA2 in renal clear cell carcinoma.

### Survival Analysis for Kidney Clear Cell Carcinoma (KIRC) Patients Revealed Methylation Status of HHLA2 DNA Was Associated With the Prognosis of KIRC Patients

**Figure 5:**
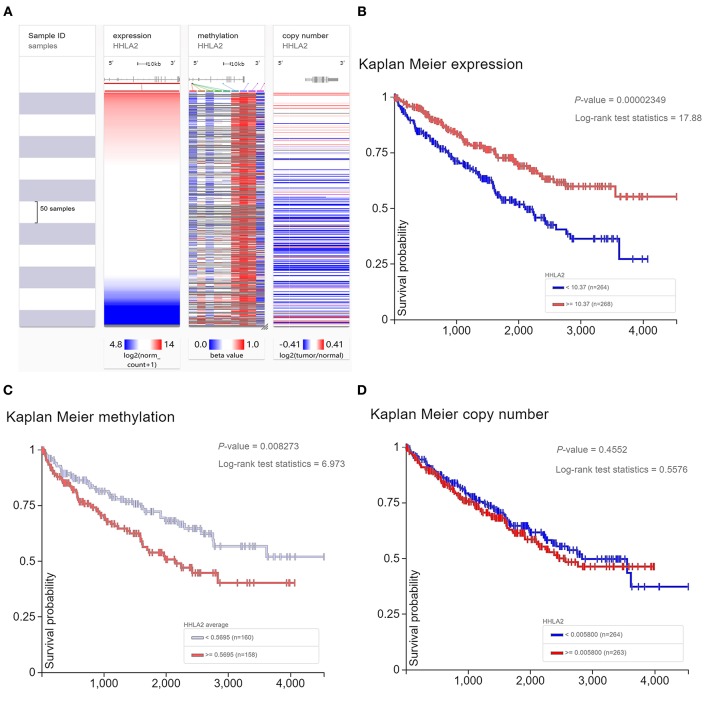
The Heatmap and Kaplan Meier curves of expression level, methylation status, copy number variant of HHLA2. **(A)** The heatmap shows the expression level, methylation status, copy number variant of HHLA2 in the TCGA database, determined by UCSC Xena. **(B–D)** Kaplan Meier plot shows the over-expression and hypomethylation of HHLA2 is favorable prognostic factor for KIRC patients.

The previous studies showed that copy number variant of HHLA2 DNA may be involved in the dysregulation of HHLA2 expression level in breast cancer (44). So, we will investigate whether DNA variant of HHLA2 have a similar effect on KIRC patients. Our result shows that the expression of HHLA2 was correlated with the methylation status of HHLA2 DNA in KIRC, but not copy number variant (Figure 5A). Then, we investigate whether DNA change was correlated with the prognosis of KIRC. Survival analysis showed that the hypomethylation of HHLA2 DNA and overexpression of HHLA2 mRNA were favorable prognostic factors for KIRC patients. However, there was no significant correlation between HHLA2 copy number variant and patient prognosis (Figures 5B–D).

### Functional Annotation of Co-expression Gene Network of HHLA2

**Figure 6:**
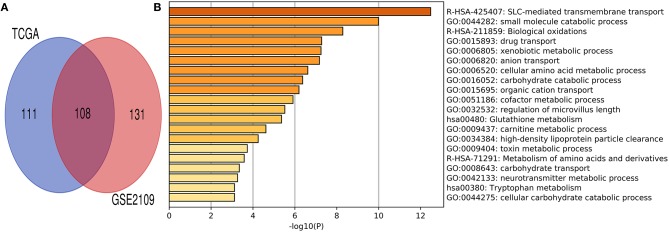
Venn plot and Metascape enrichment result of co-expression genes of HHLA2. **(A)** The intersection of Venn plot is considered to be co-expressed genes of HHLA2. **(B)** The bar plot of HHLA2 co-expression gene enrichment term.

Co-expression gene analysis is a systematic approach for analyzing the potential regulatory pattern of the complex system (45). Co-expression genes of renal clear cell carcinoma were extracted from cBioPortal and Gene expression omnibus (GEO) datasets (accession ID: GSE2109) (16). The co-expression gene of HHLA2 with the absolute value of the Pearson correlation coefficient > 0.4 was used to draw Venn plot (Figure 6A). The intersection of these gene lists was regarded as the potential co-expression gene of HHLA2 (Figure 6A, Supplementary Table 2). To investigate the potential function of co-expression genes, MetaScape (43) was used for functional enrichment. The most statistically significant terms were shown in Figure 6B. The enrichment result shows that the SLC-mediated transmembrane transport might be associated with the co-expression genes of HHLA2.

### Transcription and Post-transcription Regulation of HHLA2 Co-expression Genes

Taken together, the above finding indicated HHLA2 is significantly over-expressed in KIRC, this dysregulation may be derived from post-transcription or transcription regulation. So, we investigate what transcription factors might play an upstream regulation role of the HHLA2 co-expression network. Using the geneXplain platform, 15 transcription factors were identified (Supplementary Table 3).

Next, we further investigated whether the identified transcription factors can directly bind to the HHLA2 DNA. Chip-seq is a commonly used experimental technique for studying protein-DNA interactions (46). Cistrome data browser is a comprehensive database that integrates human and mouse transcription factors ChIp-seq, DNase-seq, and ATAC-seq experiment information. We used Cistrome data's Chip-seq data to confirm that BATF, GATA3, HSF1, HOXC9, SMAD1 can directly bind to HHLA2 DNA.

The evidence that BATF can bind HHLA2 DNA is derived from B Lymphocyte and Lymphoblastoid, SMAD1 is derived from Monocyte and Haematopoietic Progenitor Cell, and GATA3 is found in normal organs such as breast and brain. HOXC9 is found in brain and HSF1 is found in the breast. Detailed results can be found in the Supplementary Table 4. Because previous studies suggested that HHLA2 is expressed in monocyte and B lymphocytes (34, 35), so we speculate that BATF is involved in the regulation of HHLA2 expression in B lymphocytes and that SMAD1 is in monocytes.

In addition, using miRDB (38, 39), mirWalk (36, 37), and TargetScan (40) databases, we also investigated what microRNAs were involved in the post-transcription regulation of HHLA2.Using strict screening criteria (TargetScan: context++ score percentile > 98, context++ score < −0.4, miRDB: Score > 70, mirWalk: *P*-value < 0.01), two microRNAs (hsa-miR-3116 hsa-miR-6870-5p) that might bind to HHLA2 mRNA were identified (Supplementary Figure 1).

## Discussion

HHLA2 is a newly identified gene of the B7/CD28 family. Previous studies have reported that it was widely expressed in patients with PD-1-negative NSCLC, which suggests HHLA2 might be promising immunotherapy target for tumor patients who do not response to PD-1 related therapy (15). Our results are consistent with the previous reports that HHLA2 is widely expressed in a number of human tumors including kidney, Colon et al. Overall, according to expression profiling analysis of Oncomine and TCGA, HHLA2 expression was significantly elevated in most of the kidney cancers associated studys and decreased in colorectal cancer datasets.

In the terms of prognostic role, since the over-expression of immune checkpoint stimulator was always associated with a better survival time of tumor patients, these two opposite conclusions about HHLA2 function make the exact prognostic role of HHLA2 still uncertain.

Our study indicated that HHLA2 was a significant prognostic factor in a portion of tumors, but obvious heterogeneity prognostic value was observed on different kinds of tumors, part of them are protective and others are unfavorable or no significant prognostic factors.

Interestingly, a unique prognostic role of HHLA2 was observed in renal clear cell carcinoma compared with other tumor types. In a further study with KIRC as a case, we found that overexpressed or hypomethylation of HHLA2 is a favorable prognostic factor for KIRC, however, there was no significant correlation between copy number variant of HHLA2 and the prognosis of KIRC patients. Our finding was not consistent with previous report that elevated expression of HHLA2 may be associated with abnormal copy-number variant of HHLA2 DNA in basal breast cancer (44). We are unable to reconcile this discrepancy due to the lack of information on the primary antibody applied in that study.

In general, HHLA2, as a newly identified immune checkpoint molecule, little is known about the prognostic significance of HHLA2, recent publishing studies were still on “preliminary research stage” and “clinical validation stage” (47, 48), further evidence from *in vivo* and *in vitro* experimental evidence is needed for the understanding of the immune function of HHLA2, before this experimental evidence, the prognostic role should be discussed in detail, this was directly associated with the next research direction of HHLA2 and whether it is a promising prognostic marker or a new immunotherapeutic target.

According to our results, we proposed some inspiring insights and hypothesis for the research of HHLA2.

- Our result revealed that the prognostic significance of HHLA2 was with slight evidence in portion of tumors. Although some of the previous studies reported significant prognostic value of HHLA2 in a variety of tumors (47, 48), due to the lack of blind method, relatively small sample size and subjective quantitative method, high risk of bias may exist in part of the previous reports. Therefore, in the future, more rigorous researches and meta-analysis with high-qualified evidence are needed to determine the prognostic value of HHLA2, especially in KIRC, and whether it is correlated with tumor types.
- Our results showed that HHLA2 was a favorable prognostic marker for KIRC with strong evidence, however, the favorable role was not always observed in previous studies. Our finding was consistent with the first biological experimental report about the function of HHLA2 (16), but was contrary to part of previous researches (47, 48), so, why is the favorable prognostic value of HHLA2 unique and evident for KIRC? Further studies which are focused on the correlation between characteristic of KIRC and the function of HHLA2 will contribute to the understanding of the heterogeneous immune function of HHLA2.
- Gene expression dysregulation always corresponds to an important biological function in most cases. According to our result, HHLA2 played an evident protective prognostic role and was significantly overexpressed in KIRC, the biological mechanism behind this phenomenon is needed to note, further *in vivo* and *in vitro* experiment focused on this phenomenon may be helpful to understand how HHLA2 is interacting with tumor cells. In addition to the function of HHLA2 in KIRC, the potential mechanism involved in dysregulated expression of HHLA2 is also noteworthy, our result provided a possible explanation for this question, inconsistent with the previous report on breast cancer (44), the epigenetic modification but not copy number variant may be responsible for the dysregulated expression of HHLA2 in KIRC.

Our study is the first to report the potential transcriptional regulation mechanism of HHLA2 in bioinformatic view. Our result showed that BATF in B lymphocyte and SMAD in monocytes might be responsible for the dysregulation of HHLA2 in KIRC. Further studies of the BATF/HHLA2 axis and the SMAD/HHLA2 axis may help to further understand the role of HHLA2 in the human immune system.

## Data Availability

The datasets used and/or analyzed during the current study are available from the corresponding author on reasonable request.

## Author Contributions

ML and ZR drafted the paper. BW analyzed the data. YO designed the study and rephrased the paper. The paper was approved by all authors.

### Conflict of Interest Statement

The authors declare that the research was conducted in the absence of any commercial or financial relationships that could be construed as a potential conflict of interest.
