# Supplementary material for: Prognostic Significance of Potential Immune Checkpoint Member HHLA2 in Human Tumors: A Comprehensive Analysis
Source: Front Immunol. 2019 Jul 15;10:1573. doi: 10.3389/fimmu.2019.01573 (PMC6644528; doi:10.3389/fimmu.2019.01573)
Supplement: Supplementary file 1 [file Data_Sheet_1.docx]

| Cancer | HR（95%CI） | N | P-value |
| --- | --- | --- | --- |
| kidney renal clear cell carcinoma | 0.46[0.34-0.63] | 530 | 3.00E-07 |
| liver hepatocellular carcinoma | 1.93[1.36-2.74] | 371 | 0.00018 |
| pancreatic ductal adenocarcinoma | 1.98[1.21-3.24] | 177 | 0.0056 |
| thymoma | 0.16[0.03-0.8] | 119 | 0.011 |
| sarcoma | 0.62[0.42-0.93] | 259 | 0.019 |
| esophageal carcinoma | 1.77[1.06-2.97] | 161 | 0.027 |
| Breast cancer | 0.7[0.51-0.96] | 1090 | 0.028 |
| Head-neck squamous cell carcinoma | 1.38[1.03-1.85] | 500 | 0.03 |
| kidney renal papillary cell carcinoma | 0.55[0.3-1] | 288 | 0.047 |
| Bladder carcinoma | 0.72[0.51-1] | 405 | 0.05 |
| uterine corpus endometrial carcinoma | 1.7[0.96-3.01] | 543 | 0.065 |
| rectum adenocarcinoma | 0.48[0.22-1.07] | 165 | 0.067 |
| cervical squamous cell carcinoma | 0.66[0.4-1.1] | 304 | 0.11 |
| stomach adenocarcinoma | 1.31[0.93-1.83] | 375 | 0.12 |
| ovarian cancer | 0.83[0.63-1.09] | 374 | 0.17 |
| lung adenocarcinoma | 0.82[0.61-1.12] | 513 | 0.21 |
| lung squamous cell carcinoma | 1.18[0.9-1.54] | 501 | 0.24 |
| pheochromocytoma and paraganglioma | 0.37[0.07-2.04] | 178 | 0.24 |
| thyroid carcinoma | 0.55[0.2-1.52] | 502 | 0.24 |
| testicular germ cell tumor | 0.4[0.05-3.04] | 134 | 0.36 |

Table.1 The detailed result of survival analysis from Kaplan Meier plotter.

| Correlated Gene | Cytoband | Spearman's Correlation | p-Value | q-Value |
| --- | --- | --- | --- | --- |
| TMEM82 | 1p36.21 | 0.632983959 | 8.28E-41 | 1.67E-36 |
| SLC23A3 | 2q35 | 0.629631739 | 2.85E-40 | 2.88E-36 |
| SLC39A5 | 12q13.3 | 0.594030739 | 5.98E-35 | 4.02E-31 |
| BBOX1 | 11p14.2 | 0.591675086 | 1.28E-34 | 6.43E-31 |
| SLC22A7 | 6p21.1 | 0.585153994 | 1.01E-33 | 4.06E-30 |
| LGALS2 | 22q13.1 | 0.581639835 | 3.00E-33 | 1.01E-29 |
| RBP5 | 12p13.31 | 0.575080727 | 2.23E-32 | 5.82E-29 |
| FUT6 | 19p13.3 | 0.574972321 | 2.31E-32 | 5.82E-29 |
| ASPDH | 19q13.33 | 0.570413102 | 9.06E-32 | 2.03E-28 |
| APOM | 6p21.33 | 0.551121093 | 2.35E-29 | 4.74E-26 |

Table.2 Top 10 significant (q-value) co-expression genes of HHLA2 identified in all kinds of tumors. The data of spearman’s correlation coefficient, P and Q value was from kidney clear cell carcinoma.

| Transcription factors | Cutoff |
| --- | --- |
| B-ATF.1 | 0.856643 |
| CP2-L1.1 | 0.928108 |
| ERR2.1 | 0.792793 |
| GATA-3.1 | 0.808 |
| HNF-4gamma.1 | 0.86029 |
| HSF1.1 | 0.815092 |
| HTF-4.1 | 0.87037 |
| Hox-C9.1 | 0.835 |
| LRH-1.1 | 0.803804 |
| NGFI-B.1 | 0.9005 |
| SMAD1.1 | 0.874189 |
| SMAD2.1 | 0.9295 |
| SREBP-2.1 | 0.840659 |
| Tal-1; GATA-1.1 | 0.826347 |
| ZEB1.1 | 0.8755 |

Table.3 Transcription factors prediction by geneXplain platform.

| GSM | bs1 | bs2 | bs3 | IP |
| --- | --- | --- | --- | --- |
| GSM986067 | MCF-7 | Epithelium | Mammary Gland | GATA3 |
| GSM935567 | SH-SY5Y | Neuron | Bone Marrow | GATA3 |
| GSM1010738 | SK-N-SH | Neuron | Brain | GATA3 |
| GSM1680102 | BE2-C | None | None | GATA3 |
| GSM1697881 | Jurkat | T Lymphocyte | Blood | GATA3 |
| GSM1975913 | Jurkat | T Lymphocyte | Blood | GATA3 |
| GSM986069 | MCF-7 | Epithelium | Mammary Gland | GATA3 |
| GSM1010738 | SK-N-SH | Neuron | Brain | GATA3 |
| GSM1010738 | SK-N-SH | Neuron | Brain | GATA3 |
| GSM1010783 | MCF-7 | Epithelium | Mammary Gland | GATA3 |
| GSM1010738 | SK-N-SH | Neuron | Brain | GATA3 |
| GSM1010738 | SK-N-SH | Neuron | Brain | GATA3 |
| GSM1010738 | SK-N-SH | Neuron | Brain | GATA3 |
| GSM935567 | SH-SY5Y | Neuron | Bone Marrow | GATA3 |
| GSM1602666 | KELLY | None | None | GATA3 |
| GSM1680102 | BE2-C | None | None | GATA3 |
| GSM1010738 | SK-N-SH | Neuron | Brain | GATA3 |
| GSM720422 | MCF-7 | Epithelium | Mammary Gland | GATA3 |
| GSM986069 | MCF-7 | Epithelium | Mammary Gland | GATA3 |
| GSM986067 | MCF-7 | Epithelium | Mammary Gland | GATA3 |
| GSM1010783 | MCF-7 | Epithelium | Mammary Gland | GATA3 |
| GSM1241752 | MCF-7 | Epithelium | Mammary Gland | GATA3 |
| GSM1241756 | MCF-7 | Epithelium | Mammary Gland | GATA3 |
| GSM1010783 | MCF-7 | Epithelium | Mammary Gland | GATA3 |
| GSM986067 | MCF-7 | Epithelium | Mammary Gland | GATA3 |
| GSM1010738 | SK-N-SH | Neuron | Brain | GATA3 |
| GSM1010738 | SK-N-SH | Neuron | Brain | GATA3 |
| GSM1010738 | SK-N-SH | Neuron | Brain | GATA3 |
| GSM986067 | MCF-7 | Epithelium | Mammary Gland | GATA3 |
| GSM1010738 | SK-N-SH | Neuron | Brain | GATA3 |
| GSM986069 | MCF-7 | Epithelium | Mammary Gland | GATA3 |
| GSM1010783 | MCF-7 | Epithelium | Mammary Gland | GATA3 |
| GSM803538 | GM12878 | Lymphoblastoid | Blood | BATF |
| GSM803538 | GM12878 | Lymphoblastoid | Blood | BATF |
| GSM803538 | GM12878 | Lymphoblastoid | Blood | BATF |
| GSM803538 | GM12878 | Lymphoblastoid | Blood | BATF |
| GSM1370272 | OCI-Ly3 | B Lymphocyte | Bone Marrow | BATF |
| GSM1370277 | OCI-Ly10 | B Lymphocyte | Bone Marrow | BATF |
| GSM803538 | GM12878 | Lymphoblastoid | Blood | BATF |
| GSM1370277 | OCI-Ly10 | B Lymphocyte | Bone Marrow | BATF |
| GSM803538 | GM12878 | Lymphoblastoid | Blood | BATF |
| GSM1370277 | OCI-Ly10 | B Lymphocyte | Bone Marrow | BATF |
| GSM1370277 | OCI-Ly10 | B Lymphocyte | Bone Marrow | BATF |
| GSM1370272 | OCI-Ly3 | B Lymphocyte | Bone Marrow | BATF |
| GSM803538 | GM12878 | Lymphoblastoid | Blood | BATF |
| GSM803538 | GM12878 | Lymphoblastoid | Blood | BATF |
| GSM803538 | GM12878 | Lymphoblastoid | Blood | BATF |
| GSM951897 | None | Epithelium | Mammary Gland | HSF1 |
| GSM951904 | None | Epithelium | None | HSF1 |
| GSM951894 | BT-20 | Epithelium | Mammary Gland | HSF1 |
| GSM951888 | SKBR-3 | Epithelium | Breast | HSF1 |
| GSM951889 | SKBR-3 | Epithelium | Breast | HSF1 |
| GSM951895 | BT-20 | Epithelium | Mammary Gland | HSF1 |
| GSM1495182 | U2OS | None | Bone | HSF1 |
| GSM848788 | BE2-C | None | Brain | HOXC9 |
| GSM848789 | BE2-C | None | Brain | HOXC9 |
| GSM848789 | BE2-C | None | Brain | HOXC9 |
| GSM848788 | BE2-C | None | Brain | HOXC9 |
| GSM848789 | BE2-C | None | Brain | HOXC9 |
| GSM848788 | BE2-C | None | Brain | HOXC9 |
| GSM1505741 | HUES64 | Embryonic Stem Cell | Embryo | SMAD1 |
| GSM722425 | U937 | Monocyte | None | SMAD1 |
| GSM722425 | U937 | Monocyte | None | SMAD1 |
| GSM722399 | None | Haematopoietic Progenitor Cell | Blood | SMAD1 |
| GSM722399 | None | Haematopoietic Progenitor Cell | Blood | SMAD1 |
| GSM722425 | U937 | Monocyte | None | SMAD1 |
| GSM722425 | U937 | Monocyte | None | SMAD1 |
| GSM722425 | U937 | Monocyte | None | SMAD1 |
| GSM722425 | U937 | Monocyte | None | SMAD1 |
| GSM722399 | None | Haematopoietic Progenitor Cell | Blood | SMAD1 |

Table.4 Chip-seq data from Cistrome data browser.


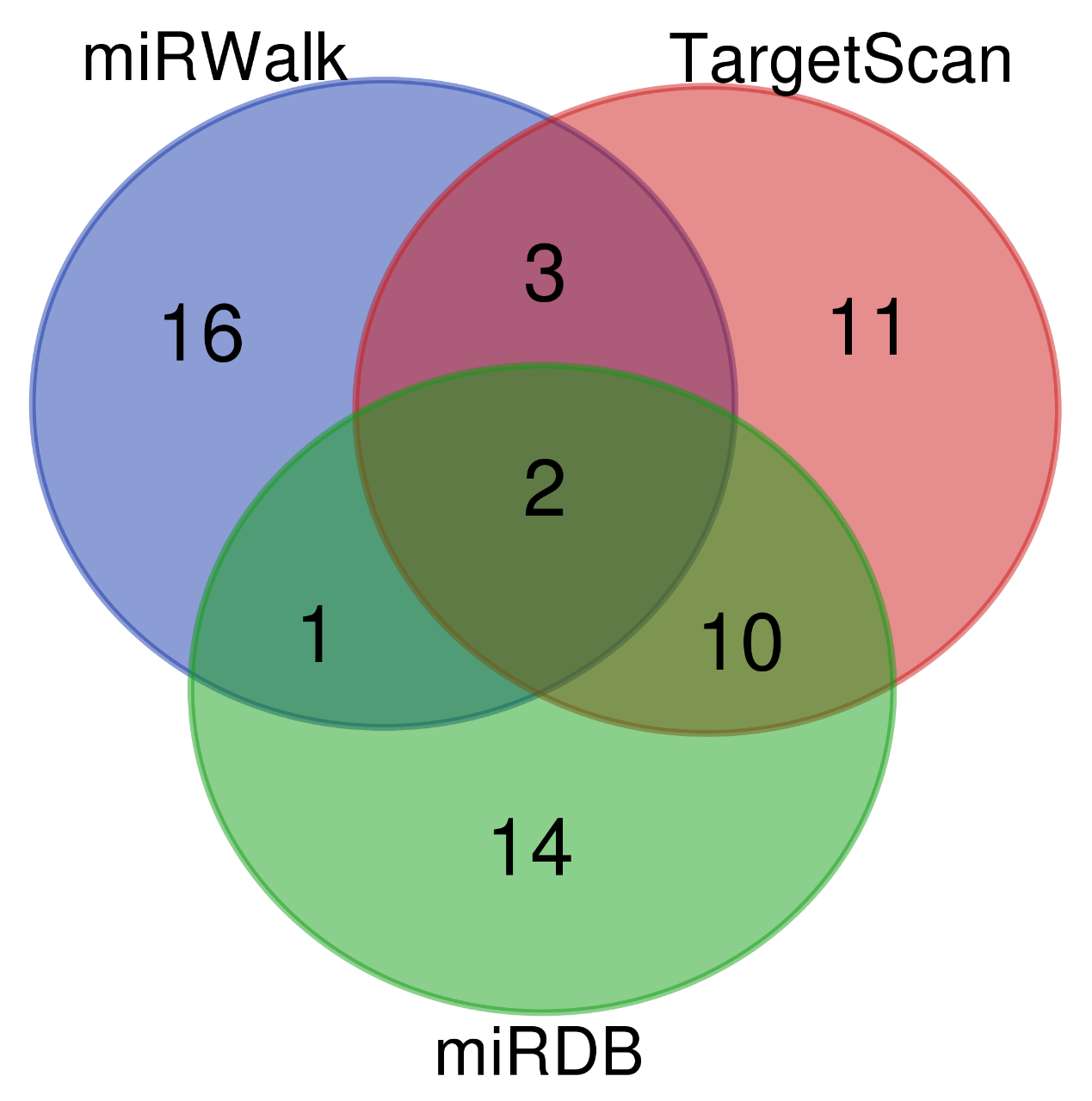


Figure.1 Venn plot of microRNA prediction result.
